# 1993. Donor-Derived Transmission of Candida species: 10-year Analysis from the OPTN ad hoc Disease Transmission Advisory Committee (DTAC)

**DOI:** 10.1093/ofid/ofad500.120

**Published:** 2023-11-27

**Authors:** Jason D Goldman, Ann E Woolley, Dong Heun Lee, R Patrick Wood, Taylor Liveli, Judith A Anesi, Gerald J Berry, Kelly E Dunn, Cynthia E Fisher, Chak-Sum Ho, Michelle Kittleson, Marty T Sellers, Sarah Taimur, Helen S Te, Anil J Trindade, Lorenzo Zaffiri, Marilyn E Levi, David Klassen, Ricardo M La Hoz, Stephanie M Pouch, Lara A Danziger-Isakov

**Affiliations:** Swedish Medical Center, Seattle, WA, USA, and Division of Allergy and Infectious Diseases, University of Washington, Seattle, WA; Brigham and Women's Hospital, Boston, Massachusetts; University of California San Francisco, San Francisco, California; LifeGift Organ Donation Center, Richmond, VA; United Network for Organ Sharing, Richmond, Virginia; University of Pennsylvania Perelman School of Medicine, Philadelphia, PA; Department of Pathology, Stanford University School of Medicine, Stanford, California; Tufts Medical Center, Boston, Massachusetts; University of Washington, Seattle, Washington; Gift of Hope Organ and Tissue Donor Network, Itasca, Illinois; Department of Cardiology, Smidt Heart Institute, Cedars-Sinai Medical Center, Los Angeles, California; DCI Donor Services, Inc., Nashville, Tennessee; Icahn School of Medicine at Mount Sinai, New York, NY; Center for Liver Diseases, University of Chicago Medicine, Chicago, Illinois; Division of Allergy, Pulmonary and Critical Care Medicine, Vanderbilt University Medical Center, Nashville, Tennessee; Division of Pulmonary Medicine, Cedars-Sinai Medical Center, Los Angeles, California; Division of Transplantation, Health Systems Bureau, Health Resources and Services Administration, Rockville, Maryland; Office of the Chief Medical Officer, United Network for Organ Sharing, Richmond, Virginia; University of Texas Southwestern Medical Center, Dallas, TX; Emory University School of Medicine, Atlanta, GA; Cincinnati Children's Hospital, Cincinnati, Ohio

## Abstract

**Background:**

Solid organ transplantation (SOT) is lifesaving, but donor-derived infections can be associated with significant morbidity and mortality. *Candida* species are common colonizers in deceased donors managed in the intensive care environment, and transmission to the recipient through transplantation is a described phenomenon. We sought to characterize *Candida* transmission events in the US SOT population.

**Methods:**

Cases referred to Organ Procurement and Transplantation Network (OPTN) Disease Transmission Advisory Committee (DTAC) between 2012 and 2022 as potential donor disease transmission events (PDDTE) were adjudicated by DTAC based on consensus definitions. We included all recipients from any donor in which ≥1 proven or probable (P/P) or possible transmission of *Candida* occurred.

**Results:**

Forty deceased donors were identified (characteristics, Table 1). 124 SOT recipients received organs from these donors including 60 kidney, 27 liver, 15 heart, 14 lung, and 8 multivisceral or other. DTAC adjudications of recipients included 8 proven, 16 probable, 24 possible, 1 unlikely, 51 excluded, and 24 intervention without disease transmission (IWDT). Growth of *Candida* in culture occurred in 24 of 40 donors and 63 of 124 recipients. Recipients were frequently bacteremic and had *Candida* growth at site of explanted kidney (Table 2). Mycotic aneurysm, bleed or hematoma occurred in 22 SOT recipients, 10 of which were P/P. Allograft explant was performed in 14 recipients (13 kidney and 1 other), 7 of which were P/P. Within 45 days of PDDTE reports, death occurred in 17 SOT recipients (1 proven, 7 possible, 7 excluded, 1 unlikely, 1 unknown). Only 6 of the 17 recipients who died received antifungal therapy. Seven deaths occurred in the 49 mate recipients of the 24 P/P transmissions, though lack of culture results did not support escalating adjudications beyond possible.

**Figure:**
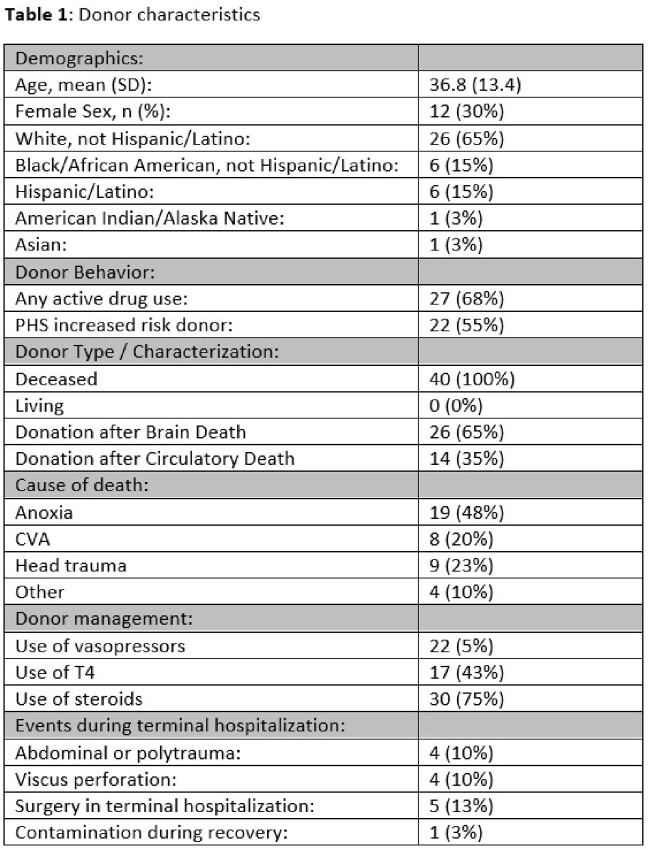

**Figure:**
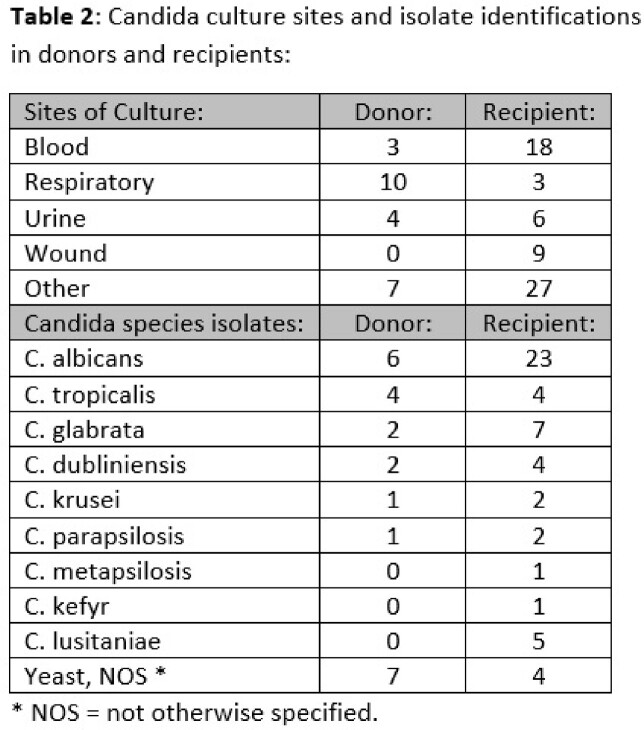

**Conclusion:**

Donor-derived Candida infections occur with significant associated morbidity, including graft loss, and mortality, especially in kidney recipients. Opportunity exists to further identify risks, improve communication across transplant centers, initiate appropriate antifungal therapy, and improve management of mycotic aneurysm due to candidiasis.

**Disclosures:**

**Jason D. Goldman, MD, MPH**, Adaptive Biotechnologies: Collaborative services agreements|Eli Lilly: Advisor/Consultant|Eli Lilly: Grant/Research Support|Eli Lilly: Honoraria|Gilead Sciences: Advisor/Consultant|Gilead Sciences: Grant/Research Support|Gilead Sciences: Honoraria|GSK: Advisor/Consultant|Karius, Inc.: Advisor/Consultant|Merck: Grant/Research Support|Monogram Biosciences / Labcorp: Collaborative services agreements|Regeneron: Grant/Research Support **Anil J. Trindade, MD**, CareDx, Inc.: Advisor/Consultant|CareDx, Inc.: Grant/Research Support|Veloxis Pharmaceuticals, Inc.: Grant/Research Support **Ricardo M. La Hoz, MD**, Takeda: Advisor/Consultant **Lara A. Danziger-Isakov, MD, MPH**, Aicuris: Contracted Clinical Research|Ansun Biopharma: Contracted Clinical Research|Astellas: Contracted Clinical Research|GSK: Advisor/Consultant|Merck: Advisor/Consultant|Merck: Contracted Clinical Research|Pfizer: Contracted Clinical Research|Roche Diagnostics: Advisor/Consultant|Takeda: Advisor/Consultant|Takeda: Contracted Clinical Research

